# Advances in Understanding Spontaneously Occurring Melanoma in Animals

**DOI:** 10.3390/vetsci10030210

**Published:** 2023-03-10

**Authors:** Kelly L. Blacklock, Louise van der Weyden

**Affiliations:** 1 Hospital for Small Animals, Royal (Dick) School of Veterinary Studies, University of Edinburgh, Easter Bush Campus, Midlothian EH25 9RG, UK; 2 Wellcome Sanger Institute, Wellcome Genome Campus, Hinxton, Cambridge CB10 1SA, UK

Melanoma is a tumour that arises from the uncontrolled proliferation of melanocytes (pigment-producing cells) found in the skin (cutaneous melanoma and digital melanoma), mucosal surfaces (oral melanoma), and the eye (ocular melanoma). Although these subtypes are all derived from melanocytes, the aetiology, pathogenesis, genetic landscape, signalling pathways and biological behaviour of each subtype are very different. There are numerous case reports of animals that spontaneously develop melanoma, ranging from fish and reptiles to birds and mammals [1]. There has also been significant research into the most commonly occurring forms of melanoma that affect dogs (oral melanoma) and cats (diffuse iris melanoma). To explore all facets of our knowledge of melanoma in animals, the Special Issue entitled “*Advances in Understanding Spontaneously Occurring Melanoma in Animals*” investigates this topic through 11 published articles; case reports, reviews and research articles all focussed on investigating the different types of melanoma in both domestic and non-domestic animals.

There are two case reports: one describes the pathology of metastatic melanoma in a lioness and the other reports on the effectiveness of multimodal treatment in a dog with malignant melanoma. The report of a cutaneous malignant melanoma on the ear of a white African lioness (*Panthera leo*) that was only diagnosed once metastasis to the liver and omentum had occurred, provides details of the clinical presentation, gross pathology and histopathological findings observed in this case and serves to show that although rarely reported in non-domesticated felids, cutaneous melanoma should be considered as a differential diagnosis for any skin mass [2]. The report of oral (lingual) malignant melanoma in a domestic dog provides a glimmer of hope for patients with this aggressive tumour type—a combination of immunotherapy and tyrosine kinase inhibitors resulted in a reduction of tumour size and the patient was still alive at 15 months post-diagnosis, with a preserved quality of life [3].

There are seven reviews which cover a variety of topics, including chromatophoromas in reptiles, melanocytic neoplasms in canines, oral melanoma in canines, feline diffuse iris melanoma and the use of the melanoma vaccine, Oncept. Chromatophoromas are a group of neoplasms arising from pigment-bearing cells (chromatophores) in the skin of reptiles and include melanophoromas (melanomas), iridophoromas, and xanthophoromas. The review by Monahan and colleagues comprehensively catalogues the occurrence and aetiology of chromatophoromas in the different species of reptiles (lizards, snakes and turtles), as well as providing clinical perspectives on their diagnosis (gross appearance, cytology, histology, immunohistochemistry and electron microscopy), staging and treatment [4]. Melanocytic neoplasms in dogs occur at a range of sites and display a variety of biological behaviours, from benign melanocytomas to malignant melanomas with high metastatic propensity, posing both a diagnostic and prognostic challenge. The review by Smedley and colleagues provides an overview of the current approaches used in the diagnosis of canine melanocytic neoplasms, discussing cytological/histological appearances (including the use of immunohistochemistry), and the current understanding of factors that affect patient prognosis, such as signalment, tumour location, specific histologic criteria, Ki-67 index, margin evaluation and lymph node assessment [5].

Malignant melanoma is the most common neoplasm of the oral cavity in dogs and is associated with poor prognosis, due to aggressive local disease, high rate of metastasis and resistance to conventional treatment therapies [6]. Kim and colleagues review the pathology of canine oral malignant melanomas and their less aggressive counterpart, histologically well-differentiated melanocytic neoplasms, encompassing signalment and presenting signs, gross appearance and cytology, histopathology, staging diagnostics, treatment options and prognosis [7]. A critical review of the treatment options available for canine oral melanomas by Pazzi and colleagues evaluates the current literature on this topic and provides treatment recommendations and suggestions for future canine oral melanoma research [8]. Hardwick reviews the somatic molecular alterations that are seen in canine oral melanomas, from single nucleotide variations to chromosomal rearrangements and dysregulation of snRNAs, and performs a cross-species comparison with human mucosal melanoma to identify recurrent aberrations and reveal biological pathways that may be critical for tumourigenesis and metastasis, with a view to considering these as potential therapeutic targets [9]. Oncept is xenogeneic DNA vaccine targeting human tyrosinase that has been USDA-approved since 2007 for the treatment of canine oral melanoma (stage II-III), as well as being used off-label for melanomas arising in other locations and species. A review by Pellin summarises the history of Oncept and evaluates the various scientific studies that have reported mixed results in terms of survival benefit in canine oral melanoma (as well as other disease sites and species) [10].

Tumours of melanocytic origin are the most common primary intraocular neoplasm of cats. The most common is feline diffuse iris melanoma, which can have a poor prognosis if not removed early, due to the risk of systemic metastasis [11]. The review by Kayes and Blacklock examines the current knowledge of feline diffuse iris melanoma, ranging from diagnostic evaluation (including a full ocular examination, iris biopsy and histopathology), factors associated with metastatic disease (such as histological characteristics, circulating cell-free DNA, and infiltrating tumour lymphocytes), and gene expression and mutational analysis [12].

There are two research articles: one is a pilot study to assess the expression of various blood biomarkers for their potential prognostic role in canine oral melanoma and the other investigates the molecular genetics of canine digital melanoma. Blood biomarkers such as lactate dehydrogenase (LDH) and peripheral leukocyte ratios are of prognostic value in human melanoma patients. The pilot study by Murray and Blacklock set out to determine if these would also prove to be prognostic biomarkers for canine oral melanoma; serum LDH levels were found to be significantly increased in dogs with oral melanoma compared to healthy controls, warranting further investigation into the benefits of using this as a prognostic biomarker [13]. In contrast to canine oral melanoma, the genetics of canine digital melanoma is relatively unexplored, so molecular investigation of this disease by Conrad and colleagues provides critical findings; assessment of the *BRAF* V595E mutation, exon 11 mutations in *c-kit*, exon 2 and 3 mutations in *KRAS* and *NRAS*, and copy number variations of *KITLG*, showed that canine digital melanoma has a distinctly different profile from that of canine oral melanoma [14].

Taken together, this Special Issue offers a rich insight into the clinical presentation, histopathology, treatment, prognosis and molecular genetics of spontaneously occurring melanoma in animals.
